# Editorial for the Special Issue on Ultrafast Laser Fabrication for Lab-on-a-Chip

**DOI:** 10.3390/mi9010038

**Published:** 2018-01-18

**Authors:** Rebeca Martínez Vázquez, Roberto Osellame

**Affiliations:** 1 Istituto di Fotonica e Nanotecnologie (IFN)—CNR, Piazza L. da Vinci 32, 20133 Milano, Italy; rebeca.martinez@polimi.it; 2 Dipartimento di Fisica—Politecnico di Milano, Piazza L. da Vinci 32, 20133 Milano, Italy

Ultrafast laser microfabrication is a very powerful method for producing integrated devices in transparent materials [1]. This technology has found applications in many diverse fields, ranging from optofluidics [2] to astrophotonics [3] and metamaterials [4]. In particular, the versatility of this technology relies on the broad portfolio of processes that it includes, encompassing subtractive (where material is removed through ablation or after selective chemical etching), additive (where material is added by two-photon polymerization), and transformative approaches (where the preexisting material is modified for waveguide writing or material welding). In this special issue, we focus on the use of ultrafast laser microfabrication for lab-on-a-chip applications, showcasing many examples where this technology has unique capabilities with respect to any other microfabrication technology, e.g., the possibility to process materials in three-dimensions.

The special issue is composed of nine papers, including original research and reviews. The different contributions can be organized into the following four categories:

(1) **Ultrafast laser ablation**. In this category, we include papers where laser ablation is used to microstructure transparent materials. Bettella et al. [5] produced the microfluidic network of a lab-on-a-chip on the surface of a lithium niobate crystal, demonstrating a droplet generator. The possibility of manufacturing microfluidic devices in lithium niobate paves the way to the realization of lab-on-a-chip devices that could exploit acoustic, electro-optic, photorefractive, and pyroelectric effects. Degawa et al. [6] used laser ablation to create miniature internal threads in a glass substrate. This component, which can only be fabricated by this technology, will prove extremely useful in the assembly of different transparent layers in the construction of a complex lab-on-a-chip device with optical access.
(2) **Femtosecond laser irradiation and chemical etching**. Here we include papers that demonstrate the potential of the combined use of femtosecond laser irradiation and chemical etching. In fact, on suitable materials (e.g., standard or porous fused silica, Foturan-Schott glasses) the irradiated pattern is selectively removed by a subsequent chemical etching step in aqueous solutions of hydrofluoric acid or potassium hydroxide. The advantage of this approach with respect to ablation is an extended 3D structuring capability and much better surface quality, as widely demonstrated by Cheng [7] in his review of high-aspect-ratio microfluidic structures. Gottmann et al. [8] showed further lab-on-a-chip devices fabricated by this approach with a particular focus on the use of potassium hydroxide wet etching, which enables unprecedented selectivity in material removal.
(3) **Two-photon polymerization**. In this category, we include papers that discuss the use of two-photon polymerization to directly write micro/nano structures with specific potential for lab-on-a-chip applications. Zandrini et al. [9] showed the use of magnetically-driven microrotors fabricated by two-photon polymerization and selective electroless plating. Such devices could be employed in lab-on-a-chip devices to actuate the flow without any external pump or as a micromixer overcoming the flow laminarity in microfluidic channels. La Fratta et al. [10] reviewed several methods used to characterize the two-photon polymerization process, and the mechanical and chemical properties of the micro/nano structures produced.
(4) **Combining subtractive and additive processes**. In this category we include papers where the above processes are combined in order to provide more functionality on the same lab-on-a-chip. Sima et al. [11] reviewed many examples of 3D micro/nano devices produced by two-photon polymerization inside microchannels fabricated by laser irradiation, followed by selective etching. Horváth et al. [12] followed the same track by specifically discussing how to compensate for aberrations when fabricating micro/nano structures by two-photon polymerization inside a sealed microchannel. Finally, Martínez Vázquez et al. [13] discussed a novel approach to develop and manufacture plastic lab-on-a-chip devices. Specifically, laser ablation on stainless steel is exploited to produce the mould for cost-effective mass-production of plastic devices by injection moulding. Femtosecond laser welding is also used to assemble the lab-on-a-chip.

We would like to thank all the contributors for submitting their papers to this Special Issue. We also thank all the reviewers for dedicating their time to help improve the quality of the submitted papers.
